# Takaysu arteritis treatment in children

**DOI:** 10.1186/1546-0096-12-S1-P355

**Published:** 2014-09-17

**Authors:** YO Kostina, GA Lyskina

**Affiliations:** 1 Pediactrics , I.M. Sechenov First Moscow State Medical University, Moscow, Russian Federation

## Introduction

Takayasu arteritis (TA) is a rare chronic inflammatory disease of unknown origin mainly involving the large vessels, such as the aorta and its primary branches. There is no uniform strategy of therapy TA in children owing to a rarity of this pathology and no specificity of clinic.

## Objectives

Efficacy of 3 treatment regiments (TR) was retrospectively analysed in 38 children with TA aged from 3 till 16 years. Follow up period was 36 mo. In 27 of 38 patients diagnosis was established after a year from emergence of the first symptoms and characterized by widespread inflammation in the aorta and its main branches.

## Methods

Group 1 (36 patients) received the combined therapy by glucocorticoids (Pr) and methotrexate (MTX). Cyclophosphamide (CYC) used at 2 patients as first therapy with widespread inflammation and in 6 – at inefficiency of primary treatment of MTX (group 2). In 5 children with refractory TA – infliximab (INF) (group 3). TR efficacy was estimated by TA activity index (AI), based on Birmingham scale of vasculitis activity (erythrocyte sedimentation rate (ESR), C-reactive protein (CRP), clinical symptoms and results of Doppler ultrasound, etc.).

## Results

1 TR was effective in 67% patients, 2 TR show reliable decrease in an index of activity in 6 months, remission is noted in 12 months therapy. The success of 3 months INF treatment was evaluated according to the normal significances of ESR, CRP and lack of active disease symptoms, after 6 months – reduced of vessel wall thickness.

Our data suggests that remission achieved in 32 patients (84%) within 2 years therapy. Full remission is reached at all patients, the diagnosis by which about 6 months from an onset of the illness and at 65% with diagnosis term were established more than 12 months.

## Conclusion

At early diagnostics and the localized type of aorta damage as starting basic therapy use of the combined therapy Pr and MTX is expedient. At MTX inefficiency, late diagnostics and widespread type of defeat of the vascular course, vazorenal arterial hypertension - should use CYC and INF.

## Disclosure of interest

None declared

